# A Course of Legal Study, Addressed to Students and the Profession Generally

**Published:** 1837-10

**Authors:** 


### PART SECOND.

### Bibliographical Notices.

---

Art. I.—*A Course of Legal Study, addressed to Students and the Profession generally.* By David Hoffman, Jur. Utr. Doct. Göttingen. Second Edition. In two Volumes.—*Baltimore,* 1836. 8vo. pp. 876. —Division vi. Medical Jurisprudence.

The only portion of this judicious and valuable work which we feel ourselves called upon to notice is the Sixth Division, on the subject of Medical Jurisprudence. The whole work is distinguished by a liberal spirit and highly enlightened views, and may be read by English as well as American students with great benefit. The course of study laid down is extensive, and it has been well observed by an American judge, that, if its precepts are steadily pursued, the next age will exhibit an American bar not excelled by any in Europe.

It seems to have been insisted on by the author, in a former publication, that ethics and law are intimately connected with physics and psychology; a philosophical view too enlarged for the mere lawyer, and which some of his American readers have received with incredulity. In the division of his work which is the subject of our remarks, he observes that Medical Jurisprudence is the connecting link between the science of law and the sciences of matter and of mind; meaning by medical jurisprudence "such a knowledge of physics and of mental philosophy as will enable legislators to enact, courts to expound, and lawyers to comprehend and safely apply laws for the preservation of life and of health; and to discover their violation, (civil or criminal,) as far as such laws are dependent upon physical or metaphysical truths." (P. 697.)

Mr. Hoffman well observes, that the legal examiner, without being of necessity a Boerhaave or a Stewart, ought to have made some acquaintance with the outlines of physical and mental philosophy, in order to become an efficient or safe adviser. In the appeal by law to medicine, "the questions can neither be propounded, nor the answers be well understood, unless the recipient, as well as the giver, have some community of knowledge, not of law, but of medicine in its largest sense." The good sense of this is so obvious, that it is a matter of surprise to find, by daily occurrences in the courts, how much it is overlooked. All the acuteness of the barrister,—all his practised readiness for getting up knowledge on short notice,—cannot always secure him from visible embarrassment when examining a medical witness, whose knowledge and whose ignorance equally baffle him; whilst the witness, blundering often from fear of making blunders, finds his best evidence sometimes misconceived, and his errors passing undetected, except by himself and the members of his own profession, who are little inclined to spare him. We think we have seen, on more than one occasion, particularly in will-cases, a trial terminate very differently from what it would have done, and ought to have done, if one or two plain and pertinent questions had been put to a medical witness. The following observations are, we think, worthy of notice by writers on this branch of knowledge.

> "There seems to be some intrinsic difficulty in executing a masterly treatise on medical jurisprudence. It should aim at nothing beyond the elucidation of doubtful legal questions, by the principles and practice of different branches of physics. If it be written by a physician, it is apt to be defective in three respects:—first, the vanity of legal authorship prompts to a too extensive, and necessarily crude, display of legal knowledge; secondly, a want of extensive acquaintance begets not only erroneous statements in law, but much that is absolutely irrelevant; and, lastly, familiarity with his own science induces too much technicality and display of learning on physical topics; so that, erroneous and inapplicable law, uniting with recondite and pedantic physics, render the work unprofitably dull. If, on the other hand, it be the production of a lawyer, it may escape the pedantry, but will scarce avoid most of the corresponding defects.
>
> "All of the treatises, indeed, in all languages, as far as we have looked at them, seem to err from the inadequate knowledge of medicine in the lawyer, or of legal science in the physician; and from the too natural desire of each to deal *learnedly* with topics foreign to his peculiar profession." (P. 699.)

One other observation we cannot but add to these remarks. We have noticed with pain in some recent publications, emanating both from the closet and the class-room, on the subject of medical jurisprudence, an elaborate indelicacy, a pruriency of expression, and a dwelling on topics which can never be quite decently exposed to the public eye. This quality has been so well appreciated by the publishers of such productions, that we have seen, in the morning papers, an advertisement of a forthcoming Lecture, bearing a heading reflecting small honour on any teacher of young men. In fact, such publications are less intended for the profession than for the public; less for instruction than for sale.

Of the works recommended by Mr. Hoffman to the American law-student, on the subject of medical jurisprudence, thirty-seven are British, six American, and thirty-three Continental; and they are selected with equal impartiality and judgment.

